# Bibliography

**Published:** 1892-03

**Authors:** 


### Bibliography.

Chart of Typical Forms of Constitutional Irregularities of the Teeth. By Eugene S. Talbot, M.D., D.D.S. Published by the Wilmington Dental Manufacturing Company. Philadelphia, 1891.

The author who has accomplished one work of real scientific value is generally satisfied to rest on the laurels earned, but Dr. Talbot goes from one point of vantage ground to another, leaving nothing undone in the search for a possible hidden fact worthy of being conveyed to his readers as a valuable lesson.

The work before us, termed very modestly, and yet appropriately, a chart, will serve to those who use it rightly as a thorough exemplification of certain typical irregular forms. We hear much of "saddle-shaped arches," "V-shaped arches," the "semi-saddle-shaped arch," etc.; but it is questionable whether the average professional man has more than an indefinite idea of what these technicalities mean.

The special life-work of Dr. Talbot seems to have been to show the importance of certain facts in the etiology of irregularities, and, if possible, to trace the origin of these various forms. The extraordinary labor which he has devoted to this, and the very considerable expenditure of means, are beyond the realization of those not familiar with the work; but probably the examples are not many of more intense devotion and persistent self-sacrifice in or outside the dental profession. The honor it has brought him has been well deserved, and while, doubtless, his labor has been made to arrive at scientific facts, it still remains for him a personal monument, to be appreciated more and more as the labor of dental science begins to approximate to the level of his investigations. That it has not yet reached that point will, probably, be acknowledged.

This chart is but another evidence of this excellent work. Here are presented in leaves, fourteen by ten inches, sixteen lithographic plates, beginning with the normal skull with a regular denture, to the "excessive development of the superior maxilla," the "arrest of development of the superior maxilla," the "arrest of development of the rami of the inferior maxilla," the "arrest of development" causing receding chin, and so on until the representations of the "V-shaped arch" and "saddle-shaped," etc., are fully and perfectly illustrated.

The original work of the artist was very superior, and while we cannot feel that these lithographic copies are equal in beauty and perfection of execution, they are very satisfactory both in design and color.

We can most cordially commend this chart as a means of instruction and study, as these figures represent types from which a great variety of modifications can be formed. Those who would make themselves familiar with these forms of irregularities of the superior and inferior maxilla can have no better guide than this chart, and by this objective lesson will be able to follow the text of this author more intelligently.

